# Correction: Structural, magnetic and hyperthermia properties and their correlation in cobalt-doped magnetite nanoparticles

**DOI:** 10.1039/d2ra90098j

**Published:** 2022-10-05

**Authors:** L. T. H. Phong, D. H. Manh, P. H. Nam, V. D. Lam, B. X. Khuyen, B. S. Tung, T. N. Bach, D. K. Tung, N. X. Phuc, T. V. Hung, Thi Ly Mai, The-Long Phan, Manh Huong Phan

**Affiliations:** Institute of Materials Science, Vietnam Academy of Science and Technology Hanoi Vietnam manhdh.ims@gmail.com; Graduate University of Science and Technology, Vietnam Academy of Science and Technology Hanoi Vietnam; Duy Tan University Da Nang Vietnam; Institute of Low Temperatures and Structure Research, Polish Academy of Sciences 50-422 Wroclaw Poland; Simulation in Materials Science Research Group, Science and Technology Advanced Institute, Van Lang University Binh Thach Ho Chi Minh City Vietnam maithily@vlu.edu.vn; Faculty of Applied Technology, School of Engineering and Technology, Van Lang University Ho Chi Minh City Vietnam; Department of Physics and Oxide Research Center, Hankuk University of Foreign Studies Yongin 17035 Republic of Korea; Department of Physics, University of South Florida Tampa FL 33620 USA

## Abstract

Correction for ‘Structural, magnetic and hyperthermia properties and their correlation in cobalt-doped magnetite nanoparticles’ by L. T. H. Phong *et al.*, *RSC Adv.*, 2022, **12**, 698–707, https://doi.org/10.1039/d1ra07407e.

The authors regret that the email address of the author Thi Ly Mai was not listed in the original manuscript. The email address of this author is maithily@vlu.edu.vn. The affiliations of this author were also incomplete. The correct affiliations are as listed above.

The Royal Society of Chemistry apologises for these errors and any consequent inconvenience to authors and readers.

## Supplementary Material

